# Supplementary material for: Resolving the full spectrum of human genome variation using Linked-Reads
Source: Genome Res. 2019 Apr;29(4):635–45. doi: 10.1101/gr.234443.118 (PMC6442396; doi:10.1101/gr.234443.118)
Supplement: Supplemental Material [file supp_gr.234443.118_Supplemental_longranger-2.2.zip › longranger-2.2.tar.bz2!/lr2.2-public/lib/go/src/code.google.com/vitess/docs/contributing/code-reviews.html]

Vitess / Code Reviews


Toggle navigation

- Overview
- Guides
- Reference
- Blog
- GitHub

- #### Overview

  - What is Vitess
  - Scaling MySQL with Vitess
  - Key Concepts
- #### Getting Started

  - Run Vitess on Kubernetes
    - Custom Docker Build
  - Run Vitess Locally
- #### User Guide

  - Introduction- Client Libraries- Backing Up Data- Reparenting
        - Schema Management
        - VSchema Guide
        - Schema Swap (Tutorial)
        - Sharding
          - Horizontal Sharding (Tutorial, automated)
          - Horizontal Sharding (Tutorial, manual)
          - Sharding in Kubernetes (Tutorial, automated)
          - Sharding in Kubernetes (Tutorial, manual)
        - Vitess Sequences
        - MySQL Server Protocol
        - Vitess and Replication
        - Update Stream
        - Row Based Replication
        - Topology Service
        - Transport Security Model
        - Launching
          - Scalability Philosophy
          - Production Planning
          - Server Configuration
          - 2PC Guide
          - Troubleshooting
        - Upgrading
- #### Reference Guides

  - Vitess API- vtctl Commands
- #### Other Resources

  - Presentations- Blog- Roadmap
- #### Contributing

  - Contributing to Vitess- GitHub Workflow- Code Reviews
- GitHub

# Code Reviews

- #### Overview

  - What is Vitess
  - Scaling MySQL with Vitess
  - Key Concepts
- #### Getting Started

  - Run Vitess on Kubernetes
    - Custom Docker Build
  - Run Vitess Locally
- #### User Guide

  - Introduction- Client Libraries- Backing Up Data- Reparenting
        - Schema Management
        - VSchema Guide
        - Schema Swap (Tutorial)
        - Sharding
          - Horizontal Sharding (Tutorial, automated)
          - Horizontal Sharding (Tutorial, manual)
          - Sharding in Kubernetes (Tutorial, automated)
          - Sharding in Kubernetes (Tutorial, manual)
        - Vitess Sequences
        - MySQL Server Protocol
        - Vitess and Replication
        - Update Stream
        - Row Based Replication
        - Topology Service
        - Transport Security Model
        - Launching
          - Scalability Philosophy
          - Production Planning
          - Server Configuration
          - 2PC Guide
          - Troubleshooting
        - Upgrading
- #### Reference Guides

  - Vitess API- vtctl Commands
- #### Other Resources

  - Presentations- Blog- Roadmap
- #### Contributing

  - Contributing to Vitess- GitHub Workflow- Code Reviews

# Code Reviews

Every GitHub pull request must go through a code review and get approved before it will be merged into the master branch.

## What to look for in a Review

Both authors and reviewers need to answer these general questions:

- Does this change match an existing design / bug?
- Is there proper unit test coverage for this change? All changes should
  increase coverage. We need at least integration test coverage when unit test
  coverage is not possible.
- Is this change going to log too much? (Error logs should only happen when
  the component is in bad shape, not because of bad transient state or bad
  user queries)
- Does this change match our coding conventions / style? Linter was run and is
  happy?
- Does this match our current patterns? Example include RPC patterns,
  Retries / Waits / Timeouts patterns using Context, ...

Additionally, we recommend every author to look over your own reviews just before committing them and check if you are following the recommendations below.
We usually check these kinds of things while skimming through `git diff --cached` just before committing.

- Scan the diffs as if you're the reviewer.
  - Look for files that shouldn't be checked in (temporary/generated files).
  - Googlers only: Remove Google confidential info (e.g. internal URLs).
  - Look for temporary code/comments you added while debugging.
    - Example: fmt.Println("AAAAAAAAAAAAAAAAAA")
  - Look for inconsistencies in indentation.
    - Use 2 spaces in everything except Go.
    - In Go, just use goimports.
- Commit message format:

  - ```
    <component>: This is a short description of the change.

    If necessary, more sentences follow e.g. to explain the intent of the change, how it fits into the bigger picture or which implications it has (e.g. other parts in the system have to be adapted.)

    Sometimes this message can also contain more material for reference e.g. benchmark numbers to justify why the change was implemented in this way.
    ```
- Comments

  - `// Prefer complete sentences when possible.`
  - Leave a space after the comment marker `//`.

During the review make sure you address all comments. Click Done (reviewable.io) or reply with "Done." (GitHub Review) to mark comments as addressed. There should be 0 unresolved discussions when it's ready to merge.

## Assigning a Pull Request

If you want to address your review to a particular set of teammates, add them as Assignee (righthand side on the pull request).
They'll receive an email.

During discussions, you can also refer to somebody using the *@username* syntax and they'll receive an email as well.

If you want to receive notifications even when you aren't mentioned, you can go to the repository page and click *Watch*.

## Approving a Pull Request

As a reviewer you can approve a pull request through two ways:

- Approve the pull request via GitHub's new code review system
- reply with a comment that contains the word *LGTM* (Looks Good To Me)

## Merging a Pull Request

Pull requests can be merged after they were approved and the Travis tests have passed.
External contributions will be merged by a team member.
Internal team members can merge their **own** pull requests.

## Internal Bug Numbers

Most of the bugs the team is working on are tracked internally.
We reference to them as `b/########` or `BUG=########` in commit messages and comments.
External users can ignore these.

Contact: vitess@googlegroups.com  **·**  
Announcements  **·**  
© 2017 Vitess powered by Google Inc
